# The Purpose of Eugenics

**Published:** 1912-09

**Authors:** 


### Department of Eugenics.

Conducted by Dr. Malone Duggan and Dr. Theo. Y. Hull.

### THE TEXAS STATE SOCIETY OF SOCIAL HYGIENE.

Headquarters: San Antonio, Texas.

DR. MALONE DUGGAN, President, San Antonio.
DR. F. E. DANIEL, 1st Vice President, Austin.
PROF. A. CASWELL ELLIS, 2d Vice President, Austin.
MRS. ELI HERTZBERG, 3d Vice President, San Antonio.
DR. THEO. Y. HULL, Secretary, San Antonio.
MR. W. L. EVANS, Treasurer, San Antonio.

EXECUTIVE COMMITTEE.

Dr. Malone Duggan, Chm.
Dr. Theo. Y. Hull, Sec'y.
Dr. W. F. McCaleb.
Hon. C. H. Jenkins.
Rev. A. W. S. Garden.
Hon. J. C. Willacy.
Mr. W. L. Evans.
Dr. Frank D. Boyd.
Dr. J. R. Nichols.
Dr. J. M. McCutchan.
Dr. W. A. King.
Dr. J. W. Torbett.
Dr. F. C. Walsh.
Dr. Joe Reuss.
Dr. Frank Paschal.

### The Purpose of Eugenics.

Galton says that the aim of eugenics is to "represent each class by its best specimens; that done, to leave them to work out their common civilizations in their own way."

By constructive and restrictive methods it seeks to eliminate the unfit from society and to increase the mental, moral and physical well-being and efficiency of the race.

It aims to elevate social and religious standards on the broad principle of the brotherhood of man and makes life more ideal and civilization greater.

It aims to accomplish its purposes by education, the no less stupendous proposition of changing the conscious attitude of civilization towards the primitive questions of personal liberty and racial responsibility, and to accomplish this by appealing to the voluntary co-operation of society rather than by repressive legislation.

